# Characterisation of the complete chloroplast genome of *Fortunella Crassifolia* Swingle and phylogenetic relationships

**DOI:** 10.1080/23802359.2019.1675554

**Published:** 2019-10-12

**Authors:** Jun-Wei Wu, Fan Liu, Na Tian, Jia-Peng Liu, Xiao-Bao Shi, Xue-Jun Bei, Chun-Zhen Cheng

**Affiliations:** a College of Horticulture, Fujian Agriculture and Forestry University, Fuzhou, PR China;; b College of Biology and Pharmacy, Yulin Normal University, Yulin, PR China

**Keywords:** *Fortunella crassifolia* Swingle, chloroplast genome, phylogenetic relationship analysis

## Abstract

In this study, we reported the complete chloroplast genome of *Fortunella crassifolia* Swingle using the HiSeq-4000 sequencing. The chloroplast genome size is 160,229 bp, which consists of a large single-copy region (87,774 bp), a small single-copy region (18,721 bp), and a pair of IR regions (26,867 bp). The chloroplast genome contains 114 unique genes, including 80 protein-coding genes, 30 tRNAs, and 4 rRNAs. Phylogenetic maximum likelihood analysis showed that *F. crassifolia* was closest to Hongkong kumquat (*F. hindsii*). The complete chloroplast genome would be subsequently used for citrus species researches.

*Fortunella crassifolia* Swingle, named as ‘Jindan’, large round kumquat, ‘Youxijingan’, ‘Ronganjingan’ in China and ‘Meiwa’ in Japan, is an important kumquat belonging to Aurantioideae. It is native to China and is mainly grown in Rongan County of Guangxi province and Youxi County of Fujian Province of China. Its fruit quality is the best among kumquats (Deng et al. 1992). The essential oil extracted from its peel is thought to be one of the best and can be used as a natural food preservative against bacteria or fungus in the food industry (Gao et al. 2012). Due to its short juvenile characteristic, *F*. *crassifolia* is recommended as suitable plant species for citrus genetic transformation studies focussing on flower and fruit development, fruit traits, etc. (Yang et al. 2007). In this study, we assembled the complete chloroplast genome of *F*. *crassifolia* using whole genome resequencing data and analysed its relationship with several citrus species.

The specimen of *F*. *crassifolia* was isolated from Zhendi village, Guanqian Town, Youxi County, Sanming city, Fujian province, China (26°11′54.74″N; 117°56′55.58″E) and samples were deposited at Fujian Agriculture and Forestry University. The genomic DNA of leaf was extracted using Plant Genomic DNA Kit (Tiangen Biotech, Beijing, CA, CHN) and stored at the Fujian Agriculture and Forestry University (No. YXJG01). Whole genome resequencing was performed on the HiSeq-4000 Sequencing platform to generate 125 bp pair-end reads (BIG, Shenzhen, CA, CHN). More than 20 Gbp high-quality reads were obtained, and clean reads were aligned to six known citrus chloroplast genomes according to Xu et al. (2019), assembled into contigs using CLC Genomics Workbench v8.0 (CLC Bio, Aarhus, Denmark), and then annotated using DOGMA (Wyman et al. 2004) and Geneious (Kearse et al. 2012). The annotated *F*. *crassifolia* chloroplast genome has been deposited in Genbank with the accession number MN495932.

The complete chloroplast genome of *F*. *crassifolia* is 160,229 bp in size, containing a large single-copy region of 87,774 bp, a small single-copy region of 18,721 bp, and a pair of inverted repeat (IR) regions of 26,867 bp. The chloroplast contains 114 unique genes, including 80 protein-coding genes, 30 tRNA genes, and 4 rRNA genes. Most of them (92) occur as a single copy, but 10 protein-coding genes (i.e. *ndhB*, *rpl2*, *rpl22*, *rpl23*, *rps7*, *rps12*, *rps19*, *ycf1*, *ycf2* and *ycf15*), 8 tRNA genes (i.e. *trnA-UGC*, *trnI-CAU*, *trnI-GAU*, *trnL-CAA*, *trnN-GUU*, *trnR-ACG* and *trnV-GAC*), and all the 4 rRNA genes (*rrn4.5S*, *rrn5S*, *rrn16S* and *rrn23S*) occur in double copies. The GC content of the chloroplast genome is 38.4% and the content of A, T, C, and G is respectively 30.5%, 31.1%, 19.6%, and 18.9%, which is almost the same as the chloroplast nucleotide constitution of *F*. *hindsii* (Xu et al. 2019).

We constructed a maximum likelihood phylogenetic tree by referring to the methods of Xu et al. (2019). Results showed that the relationships between *Fortunella* and *Citrus* species were close, and *F. crassifolia* is closest to *F. hindsii* (Figure 1). The complete chloroplast genome of *F*. *crassifolia* would provide basis for the genetic information exploration of kumquats and could be subsequently used for citrus species researches.

**Figure 1.**
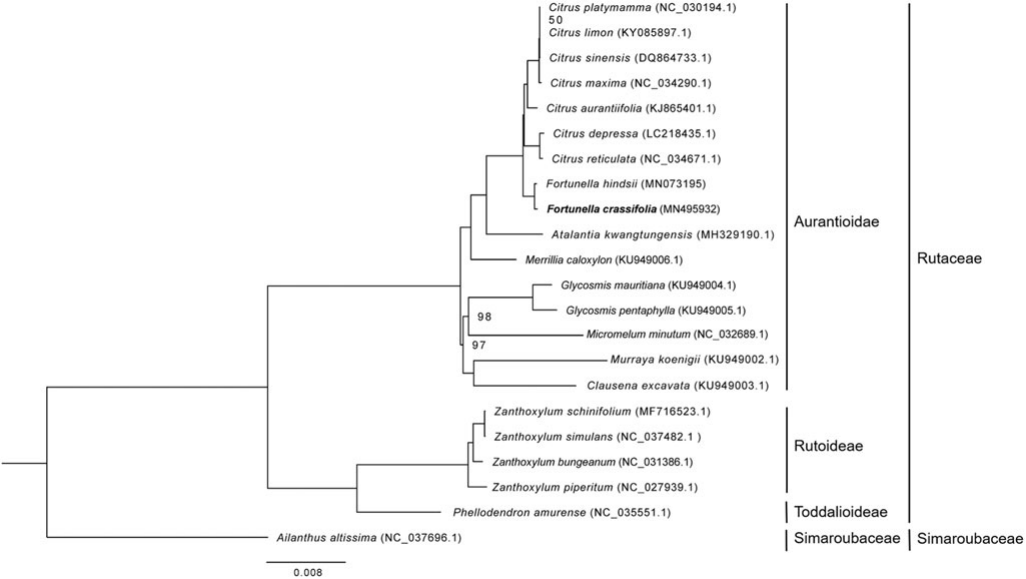
Maximum likelihood tree based on the complete chloroplast genome sequences of *Fortunella crassifolia* Swingle and 20 plant species from the Rutaceae with *Ailanthus altissima* as outgroup. Numbers shown next to the nodes are bootstrap support values based on 1000 replicates, bootstrap values of 100 were omitted.
